# Supplementary material for: Regulation of human mTOR complexes by DEPTOR
Source: eLife. 2021 Sep 14;10:e70871. doi: 10.7554/eLife.70871 (PMC8439649; doi:10.7554/eLife.70871)
Supplement: Figure 3—source data 1. — Uncropped blots of all four replicates (bands shown in Figure 3c indicated) and statistical analysis of western blot quantification. [file elife-70871-fig3-data1.pdf]

Assay replicate 1 + 2

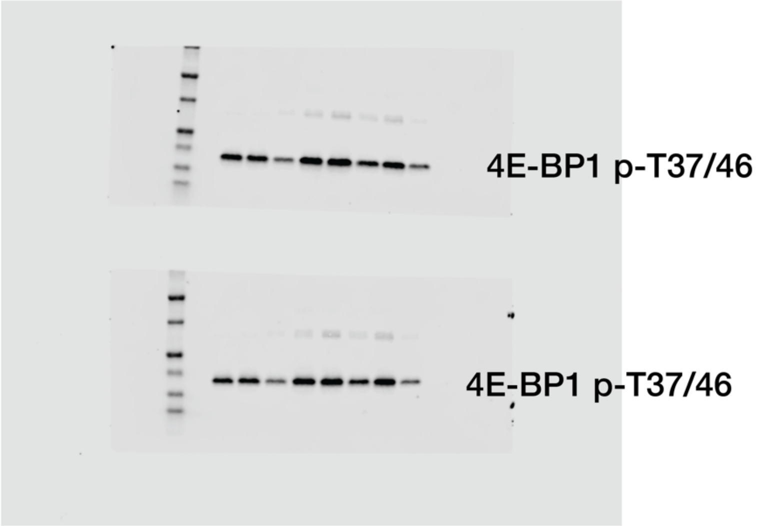

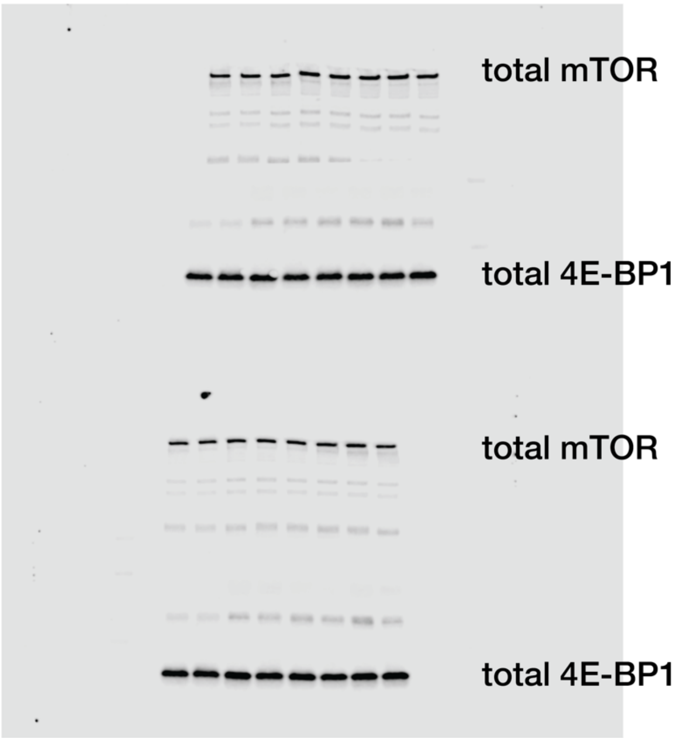

Assay replicate 3 + 4

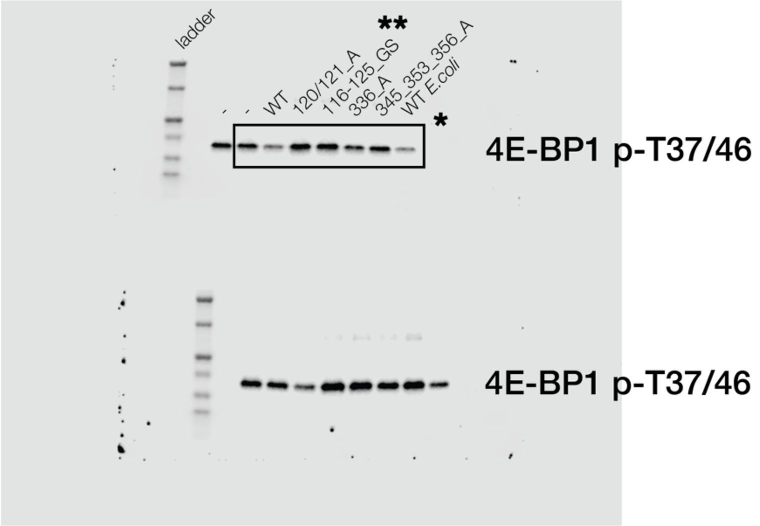

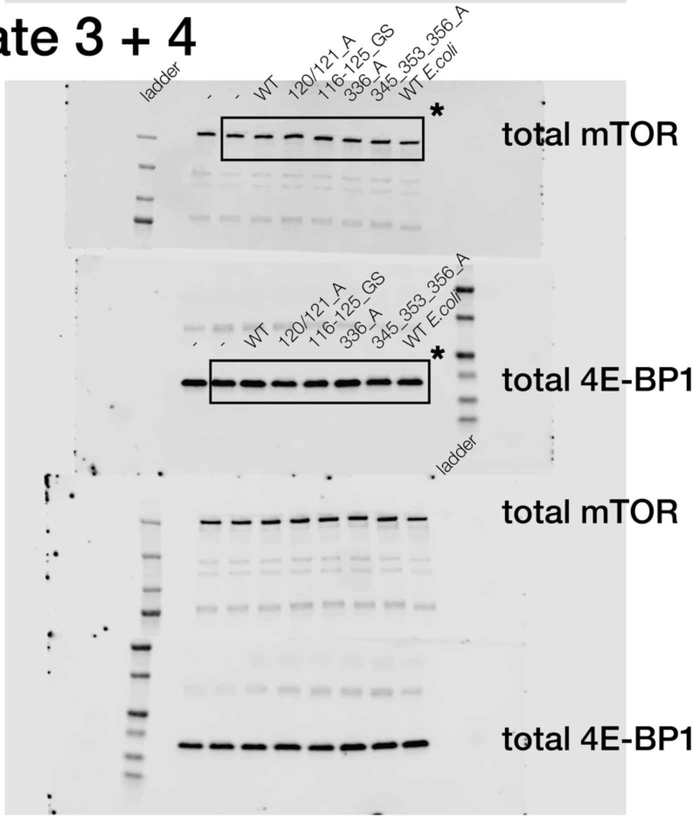

*****shown in Fig.3 c

******all replicates loaded in same order

Statistics: one-way ANOVA

Number of families	1							
Number of comparisons per family	6							
Alpha	0.05							
Dunnett's multiple comparisons test	Mean Diff.	95.00% CI of diff.	Below threshold?	Summary	Adjusted P Value	A-?		
- vs. WT	0.5981	0.3440 to 0.8521	Yes	****	<0.0001	B	WT	
- vs. 120/121_A	-0.4523	-0.7063 to -0.1982	Yes	***	0.0004	C	120/121_A	
- vs. 116-125_GS	-0.5167	-0.7708 to -0.2627	Yes	****	<0.0001	D	116-125_GS	
- vs. 336_A	0.2801	0.02603 to 0.5341	Yes	*	0.0272	E	336_A	
- vs. 345_353_356_A	-0.1262	-0.3802 to 0.1279	No	ns	0.5625	F	345_353_356_A	
- vs. WT E. Coli	0.6818	0.4278 to 0.9359	Yes	****	<0.0001	G	WT E. Coli	
Test details	Mean 1	Mean 2	Mean Diff.	SE of diff.	n1	n2	q	DF
- vs. WT	1.000	0.4019	0.5981	0.09107	4	4	6.567	21
- vs. 120/121_A	1.000	1.452	-0.4523	0.09107	4	4	4.966	21
- vs. 116-125_GS	1.000	1.517	-0.5167	0.09107	4	4	5.674	21
- vs. 336_A	1.000	0.7199	0.2801	0.09107	4	4	3.076	21
- vs. 345_353_356_A	1.000	1.126	-0.1262	0.09107	4	4	1.386	21
- vs. WT E. Coli	1.000	0.3182	0.6818	0.09107	4	4	7.487	21
