# Supplementary material for: Regulation of human mTOR complexes by DEPTOR
Source: eLife. 2021 Sep 14;10:e70871. doi: 10.7554/eLife.70871 (PMC8439649; doi:10.7554/eLife.70871)
Supplement: Supplementary file 1. [file elife-70871-supp1.docx]

**Supplementary File 1. Cryo-EM data collection and refinement statistics of DEPTOR-mTORC2 complex**

| DEPTOR-mTORC2 | | | |
| --- | --- | --- | --- |
|  | Dimer  (map1, Fig. 1-S1a) | Protomer  (map2, Fig. 1-S1a) | DEPt-Protomer  (map3, Fig. 1-S1a) |
| *Data acquisition and processing* | | | |
| EMDB accession # | 13347 | 13348 | 13349 |
| Magnification | 65,000x | | |
| Voltage (kV) | 300 | | |
| Exposure (e^-^/ Å^2^) | 50 | | |
| Frames | 40 | | |
| Defocus range (µM) | -1.0 to -3.0 | | |
| Pixel size (Å) | 1.058 | | |
| Symmetry imposed | C1 | | |
| Initial particles | 3,031,774 | | |
| Final particles | 467,078 | 750,254 | 132,837 |
| FSC resolution (masked, Å)* | 3.41 | 3.20 | 3.70 |
| *Model refinement* | | | |
| PDB ID | 7PE7 | 7PE8 | 7PE9 |
| Model resolution (Å) | 3.7/3.3 | 3.4/3.1 | 3.9/3.5 |
| FSC threshold | 0.50/0.143 | 0.50/0.143 | 0.50/0.143 |
| Bond length (Å) | 0.002 | 0.001 | 0.003 |
| Bond angle (°) | 0.440 | 0.388 | 0.535 |
| Favored (%) | 96.62 | 96.79 | 96.33 |
| Allowed (%) | 3.33 | 3.21 | 3.67 |
| Disallowed (%) | 0.05 | 0 | 0 |
| Rotamer Outliers (%) | 0.98 | 1.62 | 1.33 |
| MolProbity score | 1.43 | 1.59 | 1.68 |
| Clashscore | 4.31 | 4.43 | 6.16 |

*gold-standard FSC criterion: 0.143
