# Supplementary material for: Regulation of human mTOR complexes by DEPTOR
Source: eLife. 2021 Sep 14;10:e70871. doi: 10.7554/eLife.70871 (PMC8439649; doi:10.7554/eLife.70871)
Supplement: Supplementary file 2. [file elife-70871-supp2.docx]

**Supplementary File 2. Cryo-EM data collection and refinement statistics of DEPTOR-mTORC1 complex**

| DEPTOR-mTORC1 | | | |
| --- | --- | --- | --- |
|  | Dimer  (map4, Fig. 1-S2a) | Protomer  (map5, Fig. 1-S2a) | DEPt-Protomer  (map6, Fig. 1-S2a) |
| *Data acquisition and processing* | | | |
| EMDB accession # | 13350 | 13351 | 13352 |
| Magnification |  | | |
| Voltage (kV) | 200 | | |
| Exposure (e^-^/ Å^2^) | 50 | | |
| Frames | 40 | | |
| Defocus range (µM) | -1.0 to -2.5 | | |
| Pixel size (Å) | 0.556 | | |
| Symmetry imposed | C1 | | |
| Initial particles | 2,156,602 | | |
| Final particles | 425,076 | 850152 | 211021 |
| FSC resolution (masked, Å)* | 4.07 | 3.67 | 4.24 |
| *Model refinement* | | | |
| PDB ID | 7PEA | 7PEB | 7PEC |
| Model resolution (Å) | 6.3/4.0 | 4.3/3.6 | 4.7/4.1 |
| FSC threshold | 0.50/0.143 | 0.50/0.143 | 0.50/0.143 |
| Bond length (Å) | 0.002 | 0.002 | 0.002 |
| Bond angle (°) | 0.455 | 0.505 | 0.470 |
| Favored (%) | 95.26 | 94.86 | 95.43 |
| Allowed (%) | 4.71 | 5.09 | 4.54 |
| Disallowed (%) | 0.03 | 0.06 | 0.03 |
| Rotamer Outliers (%) | 4.24 | 2.69 | 4.99 |
| MolProbity score | 1.99 | 1.91 | 2.13 |
| Clashscore | 3.95 | 4.50 | 5.32 |

*gold-standard FSC criterion: 0.143
