# Supplementary material for: Regulation of human mTOR complexes by DEPTOR
Source: eLife. 2021 Sep 14;10:e70871. doi: 10.7554/eLife.70871 (PMC8439649; doi:10.7554/eLife.70871)
Supplement: Supplementary file 3. [file elife-70871-supp3.docx]

**Supplementary File 3. X-ray data collection and refinement for DEPTOR DEPt**

| DEPt  (PDB 7PED) |  |
| --- | --- |
| X-ray data collection | |
| Space group | P 1 2_1_ 1 |
| Cell dimensions | |
| a, b, c (Å) | 50.9, 99.0, 68.2 |
| α, β, γ (°) | 90.0, 109.7, 90.0 |
| Resolution (Å) | 99.01-1.93 (1.98-1.93) * |
| R_merge_ | 0.036 (1.41) |
| CC_1/2_ | 0.999 (0.405) |
| I/σI | 14.2 (0.6) |
| Completeness (%) | 97.4 (87.6) |
| Redundancy | 4.3 (3.1) |
| Refinement | |
| Resolution (Å) | 49.51-1.93 |
| No. reflections | 46,321(4,170) |
| R_work_/R_free_ | 0.211/0.226 |
| No. atoms | |
| Protein | 3572 |
| Water | 204 |
| B-factors | |
| Protein | 63.35 |
| Water | 56.97 |
| R.m.s. deviations | |
| Bond lengths (Å) | 0.006 |
| Bond angles (°) | 1.04 |
| Ramachandranplot |  |
| favored (%) | 98.6 |
| allowed (%) | 1.4 |
| outliers (%) | 0.0 |

*Values in parentheses are for highest-resolution shell
